# Seroprevalence and risk factors associated with Newcastle disease in backyard chickens in West Kordofan State, Sudan

**DOI:** 10.14202/vetworld.2022.2979-2985

**Published:** 2022-12-29

**Authors:** Mutaz A. I. Hussein, Nussieba A. Osman, Mohamed T. Ibrahim, Ayman M. Alhassan, Naglaa A. Abass

**Affiliations:** 1 Department of Preventive Medicine and Public Health, College of Veterinary Medicine, Sudan University of Science and Technology, Khartoum-North, Sudan; 2 Department of Clinical Sciences, Faculty of Veterinary Science, University of West Kordofan, Al-Fulah, Sudan; 3 Department of Pathology, Parasitology, and Microbiology, College of Veterinary Medicine, Sudan University of Science and Technology, Khartoum-North, Sudan; 4 Department of Animal Production, College of Animal Production Science and Technology, Sudan University of Science and Technology, Khartoum-North, Sudan; 5 Department of Poultry, Central Veterinary Research Laboratory, Soba, Khartoum, Sudan

**Keywords:** backyard chickens, epidemiology, Newcastle disease virus, Newcastle disease, risk factors, seroprevalence, Sudan

## Abstract

**Background and Aim:**

Newcastle disease (ND), a major constraint to poultry production worldwide, is a highly contagious disease of many species of domestic, exotic, and wild birds caused by ND virus (NDV). Epidemiological studies are lacking regarding ND in village chickens, including the traditional and intensive production systems used in Sudan. However, it is necessary to develop appropriate strategies to control the disease. Therefore, this study aimed to estimate the flock- and bird-level seroprevalence of NDV in backyard chickens in West Kordofan State, Sudan, and to identify the risk factors associated with ND in the study area.

**Materials and Methods:**

The seroprevalence of the circulating NDV and bird-level risk factors associated with ND was determined in backyard chickens from March to October 2017, in six villages (Alnowara, Alleait, Geibaish, Baiad, Sougoh, and Alnuhoud) in the Geibaish and Elnuhoud localities of West Kordofan State.

**Results:**

Using the hemagglutination-inhibition test, the bird- and flock-level seroprevalences of antibodies to NDV were estimated as 20.6% (78/378) and 45% (18/40), respectively. Bird-level NDV seropositivity in backyard chickens was significantly associated with the reason for raising chickens, type of housing, contact with neighboring poultry, contact with wild birds, and chicken mortality caused by infectious diseases (p ≤ 0.05).

**Conclusion:**

This study indicated that NDV is circulating in backyard chickens and may act as a potential source of infection for other birds and thus persistence of ND among local traditionally managed chickens in the areas of West Kordofan State. Risk factors contributing to ND occurrence are important for designing appropriate prevention and control strategies.

## Introduction

Poultry are generally the most abundant livestock in resource-poor areas and contribute significantly to food availability by supplying nutrient-rich and culturally acceptable products for human utilization and by enhancing crop, vegetable, and other livestock production by providing manure and pest control [1]. Traditional small-scale production from mainly indigenous stock provides the bulk of poultry output in almost all developing countries, including Sudan [2, 3], and remains the main source of dietary protein in the household [4].

Newcastle disease (ND) is caused by the avian orthoavulavirus-1 (formerly named avian paramyxovirus type-1, APMV-1), a member of the genus *Avulavirus* in the subfamily *Avulavirinae*, family *Paramyxoviridae*, and order *Mononegavirales* [5, 6]. Twenty serotypes of the APMV (1–20) have been described; however, all ND virus (NDV) isolates were identified in APMV serotype-1 [7]. Based on the fusion gene sequence, strains of APMV-1 were classified into two classes (I and II), and these classes were further classified into genotypes (I–XVIII) [8, 9].

The first records of ND in Sudan date from the 1950s and both lentogenic and velogenic NDVs were reported [10, 11]. The morbidity and mortality rates can reach 100% in outbreaks caused by velogenic strains [12]. Newcastle disease is the major constraint of village chickens in Sudan, similar to the situation in other developing countries [3, 13, 14].

No detailed epidemiological studies have been conducted in Sudan regarding ND in village chickens. To develop appropriate strategies to control the disease, epidemiological studies should be conducted in the traditional as well as intensive production systems in the country. This study was designed to estimate the seroprevalence of NDV in backyard chickens in West Kordofan State and to identify bird-level risk factors in the study area. West Kordofan State was selected as a representative for the western part of the country, where generally few studies were conducted.

## Materials and Methods

### Ethical approval

This study does not require the approval of the Institutional Ethics Committee as live animals were not used in the study.

### Study period and location

**Figure-1:**
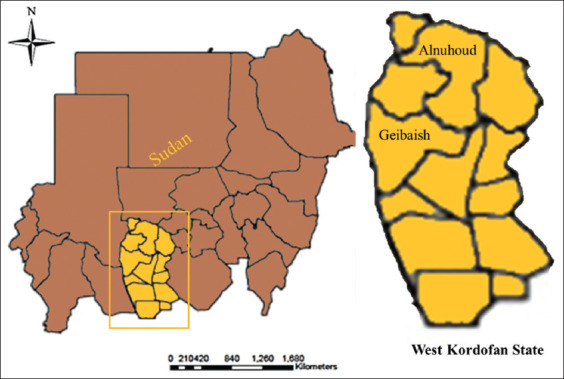
Map of Sudan showing West Kordofan State (left). The study area Alnuhoud and Geibaish localities in West Kordofan State were indicated (right) [Source: Dr. Samahir Galal Ismail Adam, Ministry of Animal Resources, Khartoum, Sudan].

We conducted a cross-sectional study from March to October 2017. This study was conducted in two localities of West Kordofan State in Sudan, namely, Elnuhoud and Geibaish (Figure-1). Elnuhoud locality covers an area of 14,496 km^2^ and lies between latitude 12–14° north and longitude 27–30° east. It has an annual level of rainfall ranging from 281 to 455 mm in the semi-desert area. Geibaish locality has an area of about 6125 km^2^ and is located at latitude 11°30’–12°30’ N and longitude 27°35’–28°30’ E. The area features a semi-desert climate with an average annual rainfall ranging from 105 to 351 mm. At the time of the study, there were no estimates of poultry population and backyard chickens in the state.

### Study population

The required sample size was based on detecting an expected NDV prevalence of 41.8% that had been previously assessed [15] with 95% confidence interval and 5% precision [16]. Therefore, the calculated sample size was estimated at 374.

In the study area, chickens are traditionally kept as free-ranging poultry by the villagers in the backyard, possibly with simple nightshades, and complete absence of veterinary services; only 21.4% of the families reared backyard chickens for their own consumption, while the majority (78.6%) raised them for income. Six villages, Baiad, Sougoh, and Alnuhoud in Alnuhoud locality and Alleait, Alnowara, and Geibaish in Geibaish locality were purposively selected for the epidemiological study because of the presence of backyard chickens (Figure-1).

A total of 378 sera were collected from the Alnuhoud (178) and Geibaish (200) localities, from chickens of >3 months of age, including unvaccinated and apparently healthy chickens. In the investigated localities, the 378 backyard chickens originally belonged to 40 flocks reared by 40 randomly selected households. All sampled chickens were of local breeds and several of them mixed with other birds and animals. Out of the 378 chickens examined for the presence of ND, 186 of these were bought from markets, whereas the remaining 192 were bought from breeders before they being raised in households.

### Sample and data collection

Blood (3 mL) was collected from the wing vein of the chicken using a sterile syringe. The collected whole blood was allowed to clot at room temperature (25°C) within the syringe and the obtained sera were stored at −20°C. For laboratory analysis, samples were transported to the Department of Poultry at the Central Veterinary Research Laboratory (CVRL), Soba, Khartoum. Blood samples were collected from investigated chickens in Baiad, Sougoh, and Alnuhoud in Alnuhoud locality and Alleait, Alnowara, and Geibaish in Geibaish locality in West Kordofan State in Sudan (Figure-1 and Table-1). Backyard chicken information was collected from villagers in all selected areas using a predesigned questionnaire. Information such as the date of sampling, owner name, location of the flock (village and locality), age of chicken, source of chicken, housing type, presence of other birds, contact with neighboring poultry and wild birds, reason for raising backyard chickens, chicken mortality, and treatment of sick birds were recorded for each bird.

**Table-1:** Seroprevalence of Newcastle disease virus in 40 flocks of backyard chickens in West Kordofan State.

Locality	Village	No. of flocks examined	No. of flocks positive	Sero-prevalence	95% exact binomial CI (%)
Geibaish	Alnowara	10	6	60	26.2–87.8
	Alleait	5	3	60	14.7–94.7
	Geibaish	5	1	20	0.5–71.6
Total		20	10	50	27.2–72.8
Alnuhoud	Baiad	4	2	50	6.8–93.2
	Sougoh	5	2	40	5.3–85.3
	Alnuhoud	11	4	36.4	10.9–69.2
Total		20	8	40	19.1–64

CI=Confidence interval

### Reference NDV and control sera

The commercial NDV La Sota vaccine strain (reference NDV strain), antiserum against NDV, and negative chicken serum used in this study were kindly provided by the Department of Poultry, CVRL, Soba.

### Hemagglutination-inhibition (HI) test

The detection of antibodies and estimation of antibody titers for NDV in chicken sera were determined for each serum sample using the HI test protocol according to the procedure described by the World Organization for Animal Health [5]. The HI titer 2^X^ (log_2_X) of serum was expressed as the reciprocal of the highest dilution causing complete inhibition of 4 hemagglutination units of the hemagglutinating NDV. Sera with titers ≥2^4^ (log_2_4) were considered positive; the validity of the results was assessed against positive and negative control sera.

### Statistical analysis

Data were analyzed using the Statistical Package for the Social Sciences version 20 after data entry using Microsoft Office Excel. Descriptive statistical analysis was first displayed in frequency distribution and cross-tabulation tables. Univariate analysis was performed using the Chi-square for qualitative data, followed by multivariable logistic regression analysis. Adjusted odds ratios (ORs) and 95% confidence intervals (CIs) were calculated to assess the strength of the association between putative risk factors and outcome variables of interest. Variables from univariate analysis with p ≤ 0.2 were included in the multivariable regression and p < 0.05 was considered statistically significant.

## Results

### Detection of NDV antibodies in backyard chicken sera

**Figure-2:**
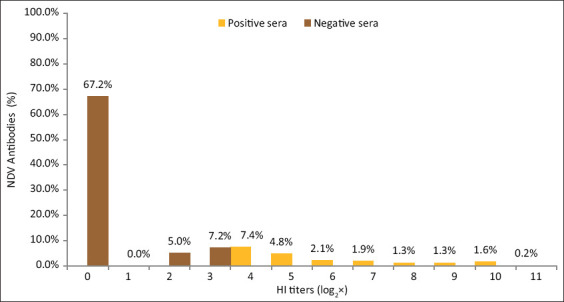
Hemagglutination inhibition (HI) test detected Newcastle disease virus antibodies in backyard chickens sera at different HI titers. Sera with HI titers ≥4 were considered as positive (yellow color), negative sera were shown (brown color).

Screening of 378 backyard chicken sera using the HI test demonstrated the presence of NDV antibodies in 78 sera (20.6%). All positive birds had a protective level of NDV antibodies due to a previous infection. The obtained HI titers of the positive sera ranged between log_2_4 and log_2_11 with an overall mean titer of log_2_1.5. However, most of the positive sera had titers of log_2_4 (28/378, 7.4%) and log_2_5 (18/378, 4.8%) (Figure-2). Based on the HI titers obtained, positive backyard chicken sera were classified as having low (ranged from log_2_4 to log_2_5), moderate (ranged from log_2_4 to log_2_7), and high (ranged from log_2_4 to log_2_11) titers (Table-2).

**Table-2:** Seroprevalence of Newcastle disease in investigated localities in West Kordofan State.

Locality	Village	No. of chickens examined	No. of chickens positive	Sero-prevalence	95% exact binomial CI (%)	HI titer	

						Total	Mean
Geibaish	Alnowara	50	14	28	16.2–42.5	89**	1.8
	Alleait	50	4	8	2.2–19.2	28*	0.6
	Geibaish	100	33	33	23.9–43.1	247***	2.5
Total		200	51	25.5	19.6–32.1	364	1.8
Alnuhoud	Baiad	50	4	8	2.2–19.2	34*	0.7
	Sougoh	50	5	10	3.3–21.8	35**	0.7
	Alnuhoud	78	18	23.1	14.3–34	141***	1.8
Total		178	27	15.2	10.2–21.3	210	1.2

Levels of HI titers:

* =Low (ranged from log_2_4 to log_2_5),

** =Moderate (ranged from log_2_4 to log_2_7),

*** =High ranged from log_2_4 to log_2_11). HI=Hemagglutination-inhibition, CI=Confidence interval

### Seroprevalence of ND in West Kordofan state

The 378 backyard chickens originated from 40 flocks in Elnuhoud and Geibaish localities in West Kordofan State. Elnuhoud and Alnowara villages had the highest number of examined flocks, 11 and 10, respectively, with five from each of the other investigated villages; Sougoh, Alleait, and Geibaish, and only four flocks from Baiad. Notably, 12.7% (48/378) of the studied backyard chickens were mixed with other birds, most of which were pigeons; 51.1% (193/378) of chickens were kept in open houses as free-ranging.

The flock-level seroprevalence of ND ranged from 20% to 60%, with an overall prevalence of 45% (18/40). All investigated village flocks had positive serum for NDV antibodies, and the highest prevalences of 60% and 50% were recorded in Geibaish and Elnuhoud localities, respectively. There was no significant difference in terms of flock prevalence between the two studied localities (χ^2^ = 0.404, p = 0.525). The prevalence of each village is presented in Table-1.

Results of the HI test demonstrated 20.6% of overall individual seroprevalence of NDV antibodies among backyard chickens. The highest individual prevalence was demonstrated in Geibaish (33%) and Alnowara (28%) villages. There was a highly significant difference in the seroprevalence of NDV in the examined chickens among different villages of the two localities (χ^2^ = 24.477, p = 0.0001) (Table-2).

### Risk factors affecting the prevalence of ND

All chickens were raised in backyards but had been sourced either from markets or from breeders (186 were from markets, whereas 192 were from breeders). Based on their source, the seroprevalence of NDV was 22.6% (42/186) among chickens from the market and 18.8% (36/192) among those from breeders. The Chi-square test showed no significant difference in seroprevalence between the two sources of chickens (χ^2^ = 0.847, p = 0.358).

Nearly 80% of the villagers raised backyard chickens for income; however, only 21.4% raised them for family consumption. The seroprevalence of NDV was relatively higher among backyard chickens raised for family consumption (28.4%, 23/81) than those raised for income (18.5%, 55/297). There was a significant difference between ND seropositivity and the purpose of raising backyard chickens (χ^2^ = 3.791, p = 0.052). Most villagers kept their birds for income compared with family consumption. Notably, chickens raised for income were less infected by NDV compared with those raised for family consumption, indicating the level of care shown by the villagers for the source of their income.

Based on the type of housing, 193 chickens were kept free-ranging, while 185 were kept enclosed. The prevalence of free-ranging chickens was 27.5% (53/193), while only 13.5% (25/185) of chickens were kept in a closed housing. The difference was significant for the types of housing (χ^2^ = 11.220, p = 0.001).

Of the 378 chickens investigated for NDV infection, only 12.7% were kept with other types of birds. Although the seroprevalence was higher in mixed flocks (27.1%, 13/48) than those with only chickens (19.7%, 65/330), no significant difference in the mean antibody levels was found between the two groups (χ^2^ = 1.396, p = 0.237).

The way of life in the study area is very simple and it is easy for the family members to observe any contact with neighboring poultry and wild birds. Among the examined chickens, a higher prevalence was recorded in those who came in close contact with neighboring poultry (27.9%, 36/129) and 31.1% (23/74) in chickens that had access to wild birds compared with other groups that had no contact history. Therefore, there was a significant difference (χ^2^ = 6.324, p = 0.012 and χ^2^ = 6.131, p = 0.013) between NDV seroprevalence and close contact with neighboring poultry and wild birds, respectively.

Based on our observations concerning their health history, the backyard chickens that experienced mortality due to infectious diseases (61.7%, 66/107) were more likely to develop ND than those without reported deaths (4.4%, 12/271). The Chi-square test revealed a highly significant association between NDV and chicken mortality due to the presence of infectious diseases (χ^2^ = 153.5, p ≤ 0.001).

The majority (88.9%, 336/378) of the surveyed chickens received treatment when they became sick because of different infectious diseases, in addition to ND, as confirmed by their owners. Univariate analysis demonstrated that there was no significant association between NDV infection and treatment of diseased birds (χ^2^ = 0.890, p = 0.345). However, ND was more prevalent in chickens without a history of treatment (26.2%, 11/42) compared with other groups that had been treated (19.9%, 67/336). The univariate analysis of potential risk factors for ND in backyard chickens in West Kordofan State is presented in Table-3.

**Table-3:** Univariate analysis of potential risk factors for Newcastle disease in backyard chickens in West Kordofan State.

Risk factor	No. of birds examined	No. of positive birds	Prevalence (%)	χ^2^	p-value
Source of chickens					
Market	186	42	22.6	0.847	0.358
Breeder	192	36	18.8		
Reason for raising chickens					
Income	297	55	18.5	3.791	0.052
Family consumption	81	23	28.4		
Type of housing					
Open	193	53	27.5	11.220	0.001
Closed	185	25	13.5		
Presence of other birds					
Yes	48	13	27.1	1.396	0.237
No	330	65	19.7		
Contact with neighboring poultry					
Yes	129	36	27.9	6.324	0.012
No	249	42	16.9		
Contact with wild birds					
Yes	74	23	31.1	6.131	0.013
No	304	55	18.1		
Chickens mortality due to infectious diseases					
Yes	107	66	61.7	153.5	0.001
No	271	12	4.4		
Treatment of poultry if they are sick					
Yes	336	67	19.9	0.890	0.345
No	42	11	26.2		

Six variables were considered in the final logistic regression model (Table-4) and only “Chicken mortality due to infectious diseases” was found to be positively associated with ND viral infection (OR: 40.4, 95% CI: 18.783–86.826, p ≤ 0.001).

**Table-4:** Result of multivariable analysis of potential risk factors for Newcastle disease in backyard chickens in West Kordofan State.

Risk factor	Odds ratio	95% CI	p-value
Reason for raising chickens	0.302	0.130–0.702	0.005
Type of housing	1.39	0.511–3.798	0.520
Contact with neighboring poultry	1.44	0.565–3.679	0.440
Contact with wild birds	1.2	0.494–2.865	0.699
Chickens mortality due to infectious diseases	40.4	18.783–86.826	0.001
Presence of other birds	1.69	0.277–1.715	0.420

CI=Confidence interval

## Discussion

The results of the recent investigation in six villages (Alnowara, Alleait, Geibaish, Baiad, Sougoh, and Alnuhoud) in Geibaish and Alnuhoud localities of West Kordofan State revealed a relatively high serological prevalence of NDV of 45% (29.3%–61.5% at 95% CI) among the backyard chicken flocks. Alnowara, Alleait, Geibaish, Baiad, Sougoh, and Alnuhoud had flock prevalence of 60%, 60%, 20%, 50%, 40%, and 36.4%, respectively, with no significant difference (χ^2^ = 0.404, p = 0.525) in antibodies to NDV flock seroprevalence at the locality level. The presence of a high prevalence of NDV antibodies in seropositive flocks may be caused by the circulation of low pathogenic NDV strains producing no clinical signs in these birds, as has been stated previously [17].

Examination of sera from the 378 backyard chickens revealed that 78 sera (20.6%, 16.7%–25.1% at 95% CI) were positive for NDV antibodies with a highly significant difference in ND individual seroprevalence among the different investigated villages. Based on our observations, the high NDV seroprevalence in Geibaish locality and bordering Darfur States may be attributable to the high number of wild birds and poor management practices of traditionally kept backyard chickens compared with those features in Alnuhoud locality bordering North Kordofan State. As there was no history of vaccination against NDV in the study area, the reported seroprevalence in apparently healthy birds was considered as clear evidence of exposure of these chickens to NDV, possibly through contact with infected birds. Positive serology in unvaccinated birds is considered as diagnostic evidence for the presence of ND [18]. Newcastle disease has been reported in traditionally managed village poultry in different areas within the country. In a previous study, serum samples were taken from 910 non-vaccinated birds (843 chickens, 45 pigeons, and 22 ducks) kept under the backyard management system in 14 states of Sudan [15]. Hemagglutination inhibition antibodies against NDV were detected in 41.8% of chickens with a mean antibody titer of log_2_2.75, considering that none of the states investigated were found to be free from NDV antibodies. The Western states reported a disease prevalence of 37.5% in West Darfur, 37.7% in North Kordofan, 38.9% in South Kordofan, and 41.3% in South Darfur; however, in this survey, we detected a much lower individual prevalence (20.6%) of ND in West Kordofan State.

In similar studies, variable seroprevalence values had been reported elsewhere. For instance, 26.8% seroprevalence of NDV antibodies was reported in unvaccinated backyard poultry in Bahi and Njombe districts of Tanzania [19], whereas in Nigeria, 57% of the total serum samples collected were positive for antibodies to NDV [20]. An overall NDV seroprevalence of 30% has been reported in selected districts of Buno Bedele Zone, Ethiopia, [21].

In this study, HI titers of log_2_4 and above were generally considered as positive for antibodies to NDV [22, 23]. In the two localities studied, the HI titers for the positive samples ranged from log_2_4 to log_2_11 with an overall mean titer of log_2_1.5. However, most of the sera had titers of log_2_4 and log_2_5. The higher NDV antibody titers are indicative of recent infection and may be attributed to the fact that natural infection of chickens with NDV produces higher antibody titers than vaccination [23, 24].

In a risk factor investigation, although there was no significant difference in the seroprevalence of ND between the sources of chickens, it was clear that the disease is more prevalent in chickens sourced from the market. This finding is logical as the bird market is an important source of infection where infected birds can simply mix with susceptible ones. Haile *et al*. [25] recently investigated the role of live chicken markets in the maintenance and spread of ND in the village chickens and proved that apparently healthy appearing birds were reservoirs of velogenic NDV strains that could initiate the endemicity of ND cycles in the village setting due to ease of contact at local open-air markets. Interestingly, chickens raised for income were less infected by NDV compared with those raised for family consumption; this shows that villagers care more about the source of their income.

The infection rate of the disease was 2-fold higher in free-ranging backyard chickens. The free-ranging production system increases the chance of contact with wild birds, and consequently, free-ranging chickens may serve as a threat to wild birds and other poultry and vice versa. Moreover, contact was reported for approximately 34.1% (129/378) and 19.6% (74/378) of the examined backyard chickens with neighboring or wild poultry, respectively, suggesting the spread of ND within village flocks. In this study, contacts with both neighboring poultry and wild birds were considered as risk factors associated with the occurrence of ND among the investigated backyard flocks. This result is consistent with a previous report and confirms that backyard poultry was significantly affected by close contact with wild birds [26].

In this study, mortality was reported for 28.3% of the investigated birds (107/378) because of infectious diseases and ND was more prevalent in chickens with a record of mortality (61.7%). Exposure of traditionally managed backyard chickens to infectious diseases, and therefore mortality, suggested that those birds were kept under inadequate healthcare, which increases the likelihood of contracting diseases resulting in severe economic losses.

Our results indicated that ND was prevalent in the study area throughout the study period. Newcastle disease is reported to occur throughout the year in rural poultry populations in most countries, specifically, those in Africa [27, 28], and the incidence of ND is associated with periods of climatic stress.

## Conclusion

This study provides evidence of a relatively high serological flock prevalence of NDV in backyard chickens in West Kordofan State in West Sudan. The bird seroprevalence of ND among the examined unvaccinated chickens could be attributed to different factors: Absence of vaccination, poor management system, contact with wild birds and other poultry, and inadequate healthcare. As almost 80% of the interviewed owners raised chickens for income, it is recommended that chicken owners should be aware of the economic significance of the disease and be educated on biosecurity measures. Future studies should be planned to cover other areas of the country to include a large number of backyard chickens and different types of birds and to consider additional risk factors that may be associated with the disease.

## Authors’ Contributions

MAIH: Performed field investigations, sample collection, laboratory work, and data analysis. NAA and NAO: Supervised the study, designed the work, performed data analysis and interpretation, and prepared and finalized the manuscript. MTI: Performed statistical analysis. AMA: Performed laboratory work with MAIH. All authors have read and approved the final manuscript.
